# The Impact of Career Competence on Career Sustainability Among Chinese Expatriate Managers Amid Digital Transformation in Vietnam: The Role of Lifelong Learning

**DOI:** 10.3389/fpsyg.2022.791636

**Published:** 2022-03-03

**Authors:** Wei Zhang, Tachia Chin, Fa Li, Chien-Liang Lin, Yi-Nan Shan, Francesca Ventimiglia

**Affiliations:** ^1^College of Business, Honghe University, Mengzi, China; ^2^College of Science and Technology, Ningbo University, Cixi, China; ^3^Faculty of Economics, University of Rome “Niccolò Cusano,” Rome, Italy

**Keywords:** career sustainability, lifelong learning, expatriate, mid-level managers, career competencies

## Abstract

Digitalization and advanced technologies are replacing human jobs. Around the world, many people have lost their jobs due to increasing digitalization. Similarly, Chinese expatriates associated with the manufacturing sector in emerging countries such as Vietnam face similar challenges. Therefore, Chinese expatriates need to bring competitiveness in their competencies. This competitiveness brings sustainability to their career. The aim of this study is to investigate the impact of career competencies on career sustainability. Moreover, we test the mediating effect of lifelong learning in the relationship between career competencies and career sustainability. A questionnaire survey approach was used in this study. The target population was the Chinese expatriate managers working at China-invested manufacturing multinational organizations in Vietnam. To estimate the proposed relationships, we use structural equation modeling. The results are confirmed that in the direct relationship career competence has a positive impact on career sustainability. The findings of this study also indicate that career competencies have a positive impact on lifelong learning. Furthermore, outcomes confirmed that lifelong learning has a positive impact on career sustainability. Similarly, results are also confirmed that lifelong learning is positively mediating between career competencies and career sustainability. Therefore, the empirical results of this article identify that lifelong learning has a critical impact on sustainable careers. Specifically, this study is useful for mid-level managers who are associated with multinational organizations. At the end of this article, we also explained the practical implications, limitations, and future research directions.

## Introduction

The mobility of human resources is increasing day by day, and we cannot avoid the impact of globalization. If we make inroads overseas, it becomes essential to secure local human resources, but it is common for employees to work as expatriates to advance their careers. Moreover, multinational organizations incorporate global standards, keeping in mind the high mobility of the labor market. Similarly, Chinese MNCs are identified as the powerful emerging drivers of global economic growth (Rui et al., 2017; Song et al., 2021). Following this trend, more and more Chinese workers have been expatriated to operate factories in less-developed yet populous Southeast Asian countries such as Vietnam, Malaysia, Cambodia, and Myanmar. According to the transaction theory, assigning expatriates is the significant governance approach for MNCs to reduce the transactional costs of managing local employee managers in the host country (Tan and Mahoney, 2006). It is observed that most of the Chinese expatriates working in Vietnam are mid-level managers. Prior studies indicate that expatriate managers have gradually developed career competencies in the host countries (Chin and Rowley, 2018). However, many workers and employees associated with the MNCs are worldwide lost their jobs due to increasing digitalization (Chin et al., 2021b). Similarly, Chinese expatriates associated with the manufacturing sector organizations face such a challenge. Specifically, Artificial Intelligence (AI) is widely used in emerging countries. Some emerging countries performed the basic AI that affects job demands. Similarly, some counties used advanced technologies in the organizations to enhance competitiveness of the manager’s competencies (Chin and Liu, 2015; Chin et al., 2019a). So, the advanced competencies boost career sustainability. However, the career sustainability of mid-level expatriate managers decreases day by day. Previous studies demonstrate that advanced skills and competitive competencies enhance career sustainability (Chin et al., 2019b, 2021a).

In this regard, the overall job demands-resources relationship is a matter of great concern (Bakker and Demerouti, 2014). Digitalization in manufacturing organizations increases the job demands. Therefore, mid-level managers need to learn more knowledge and skills as critical resources to meet the high job demands. The high demands of the job indicate the importance of lifelong learning. Hence, the managers need to upgrade their competencies to meet the high job demand. There is a lot of evidence from prior studies that show a significant relationship between career competencies and lifelong learning (Akkermans and Tims, 2017; Blokker et al., 2019). However, competencies have a positive association with lifelong learning, which also positively affects career sustainability (Billett, 2010; Heslin et al., 2020). This kind of research has not been conducted in previous studies. Previous studies have only focused on career competencies and career sustainability and have ignored lifelong learning. Therefore, this is the first study in which we test the combined effect of career competencies, lifelong learning, and career sustainability, especially considering lifelong learning as a mediating variable. Therefore, the purpose of our research is to address the research gap by empirically investigating the mechanisms among career competencies, career sustainability, and lifelong learning of Chinese expatriate managers working in Vietnam. The novel contribution of this study is to test the mediating effect of lifelong learning between career competencies and career sustainability. Hence, based on the above discussion, we generate the below-mentioned three research questions (RQs):

**RQ1:** How do career competencies influence career sustainability?
**RQ2:** How does lifelong learning mediate between career competencies and career sustainability?

### Hypotheses Development

According to Akkermans et al. (2013, p. 246), career competencies are defined as “the knowledge, skills, and abilities central to career development, which can be influenced and developed by the individual.” Moreover, they suggest that most recognized career competencies include three dimensions: reflective career competencies, communicative career competencies, and behavioral career competencies. Chin et al. (2021a) adopted an interactional perspective, proposing that career sustainability involves both a property of careers and a property of the people embedded in those careers. Their scale has four dimensions: flexibility, which refers to a flexible, adaptable attitude toward continuous learning; renewability, which characterizes the extent to which careers offer the opportunity for individuals to reassess their competencies; integrative, which reflects the extent to which individuals absorb disparate knowledge that they have acquired; resourcefulness, which characterizes the importance of seeking and leveraging resources to ensure employability.

Professional psychology defines lifelong learning as a sustainable process that supports individuals to acquire values, knowledge, and skills across their occupational lifetime and use them efficiently in the working environment (Lichtenberg and Goodyear’s, 2012, p. 3). According to the definitions made by Kirby et al. (2010), lifelong learning consists of behavioral and capability components characterized by five dimensions: goal setting, application of knowledge and skill, self-direction and evaluation, locating information, and adaptable learning strategies. According to the career construction theory (CCT) (Savickas, 2005; Savickas and Porfeli, 2012), career development can be seen as a constantly adapting process of individuals to the changing work environment, whereby individuals can achieve sustainability in work and life. The CCT theory has been widely used in career competencies studies because it largely echoes the core of the JD-R framework. The employees often use their career competencies as critical resources to tackle the intensifying job demands, and for constructing their careers successfully and smoothly in a context riddled with uncertainties (Rudolph et al., 2019).

Previous studies indicate that career competencies positively and significantly impact career sustainability (Chin et al., 2019a; Ren et al., 2020). Prior studies also showed that career competencies could be regarded as valuable personal resources that help workers at different levels acquire lifelong employability (Wittekind et al., 2010; De Vos et al., 2011; Akkermans et al., 2013). Extending the arguments above, we further argue that during the transformation of technology in emerging countries, Chinese expatriate managers play a critical role. These managers also learn and transfer technological-oriented skills and capabilities among the host organizations (Iqbal et al., 2021). Specifically, the Chinese expatriate managers associated with the manufacturing organizations in Vietnam also successfully deliver technical and technological skills, and learn new skills to bring sustainability to their careers. Thus, the positive relationship of career competencies with career sustainability and lifelong learning is depicted in the following hypothesis.

**H1:** Career competencies of Chinese expatriate managers positively impact their career sustainability.
**H2:** Career competencies of Chinese expatriate managers positively impact their lifelong learning.

As discussed above, the CCT highlights the importance of lifelong learning in developing and sustaining careers in terms of adapting to the constantly changing work environment and turbulent job markets. Individuals with well-established career competencies can efficiently control career-related lifelong learning (Forrier and Sels, 2003; Kuijpers et al., 2006; Bridgstock, 2009). Following this logic, as the Chinese expatriate managers in Vietnam are concerned, the positive outcome of lifelong learning, including renewed professional knowledge and skills, may offer them competitive adaptability to sustain their careers. In other words, lifelong learning can be deemed as a career strategy for those Chinese managers to realize the goal of person-environment integration. The above discussion also proves that lifelong learning positively and significant impact on the career sustainability of individuals. Hence, the above relationship is shown in the following hypothesis.

**H3:** The lifelong learning behavior of Chinese expatriate managers positively impact on career sustainability.

**FIGURE 1:**
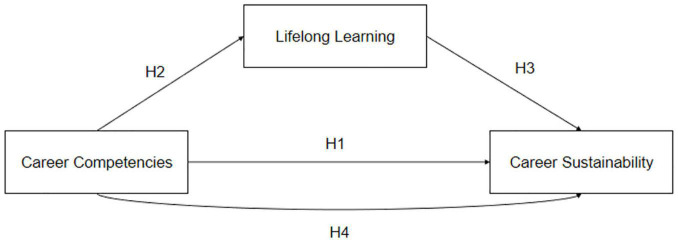
The research framework.

Previously, few studies have examined the relationship between career competencies and career sustainability using lifelong learning behavior as a mediating variable. Similarly, some studies suggest that lifelong learning enchases career sustainability and some researchers demonstrate in their studies that career competencies bring lifelong learning behavior among the workers (Fleisher et al., 1996; Parker et al., 2009). In the current situation of COVID-19, organizations are facing serious challenges that also affect individuals’ career competencies, career sustainability, and lifelong learning behaviors (Ren et al., 2020). As discussed above, the literature highlights that lifelong learning positively and significantly mediates between career competencies and career sustainability. In addition, Figure 1 presents a comprehensive research framework of this research. In this spirit, we also hypothesized the following.

**H4:** The lifelong learning behavior of Chinese expatriate managers positively mediates between the relationship of career competencies and career sustainability.

## Methods

### Research Approach

In this study, we used the online survey as the research approach, because it is common and broad level data can collect from the target population. Moreover, the data collection cost is relatively low as compared to other methods (Wang et al., 2021; Rasool et al., 2021a). In this study, first, we design the research questionnaire to collect the data.

### Instrument Designing

In this study, first, we designed a questionnaire for data collection, and the constructed hypotheses provided the base. To ensure the understanding of the questions, the survey was conducted in the Chinese language while the wordings of the questionnaire was adapted to fit the Chinese context. To enhance methodological rigor, we also used the back-translation method to ensure the accuracy of the items translated. Content validity was an essential concern in our questionnaire design (Hinkin, 1995). Therefore, to resolve this issue, we conduct a pilot study of the instrument. The pilot study respondents were six academic researchers and professors (three Ph.D. students, two associate professors, and one full professor). These researchers and professors were aware of the topic of the study. Similarly, we selected three practitioners (one HR manager in MNCs, one general manager in MNCs, and one mid-level manager) as the pilot study respondents. These respondents suggest some modifications to the research instrument. Therefore, after carefully revising the instrument, we were finalized 30 items for the instrument. Finally, we distributed the research instrument among the target population for data collection.

### Variables Measurements

In this study, we used career competencies as an independent variable and career sustainability as the dependent variable. Moreover, we used lifelong learning as a mediating variable. The independent variable career competencies were measured with the scale developed by Akkermans et al. (2013). All the career competencies items were measured using the six-point Likert scale (1 = strongly disagree to 6 = strongly agree). The same scale has been widely used in the field of management. Sample items were: “I know what I like in my work,” “I am aware of my talents in my work,” and “I know how to search for developments in my area of work.” The findings of this article demonstrate that the alpha value of career competencies is 0.938. The acceptable standard value of alpha is 0.70 or higher (Rasool et al., 2021b). Therefore, the items we were used in this study were reliable and valid.

The dependent variable career sustainability was measured with the scale developed by Chin et al. (2021a). Items of the scale have been adopted and modified. All the items of career sustainability were measured using the six-point Likert scale (1 = strongly disagree to 6 = strongly agree). Sample items include “My career allows me to continuously learn new things,” “My career enables me to critically evaluate information obtained from different sources,” and “My career makes me feel happy because I use my resources well.” The outcomes of this research point that the alpha value of career sustainability is 0.894, which is up to the acceptable standard. Therefore, the items of career sustainability were reliable and valid.

The mediating variable, lifelong learning, was measured by Kirby et al. (2010). All the items of lifelong learning were measured using the six-point Likert scale (1 = strongly disagree to 6 = strongly agree). Sample items include “I can deal with the unexpected and solve problems as they arise,” “When I approach new material, I try to relate it to what I already know,” and “I prefer to have others plan my learning.” The results of this study indicate that the alpha value of lifelong learning is 0.879, which is up to the acceptable standard. Therefore, the items we were used in this study were reliable and valid.

### Sample and Data Collection

Before the formal survey, we executed in-depth interviews with seven Chinese expatriate managers who have more than 7 years of experience working in Vietnam to ensure the properness and clearness of our hypotheses and measures. Their feedback showed that our assumptions were logical and compatible with reality. The formal survey was conducted online from June to September 2021. In this study, the Business Association of China in Vietnam^1^ supported us to collect data. The survey participants were Chinese expatriate managers working at China-invested manufacturing MNCs in Vietnam. The instrument of this study was posted on a survey website, WJX.^2^ This website is a widely accepted professional online survey service in China. When conducting the formal online survey, we explained the confidentiality of the survey process. None of the questions involved confidential information, and all the respondents were voluntary. Finally, after excluding 32 invalid questionnaires, we obtained 230 usable data.

### Demographics

The survey respondents were Chinese expatriate managers from China-invested manufacturing MNCs in Vietnam. Of the respondents’ participants, 62.17% were female and 37.83% male; about 80% were under 40 years old; 54.78% were married, and 45.22% were unmarried. Moreover, 13.91% had masters’ degrees, 59.57% had bachelors’ degrees, and 26.52% had an associated degree or below. The participants were from diversified manufacturing sectors: 21.3% from light the textile industry, 12.17% from the auto parts manufacturing, 5,65% from the machinery industry, 20.87% from the electronic manufacturing, 12.17% from automation industry, and 32.17% from original equipment manufacturing. Moreover, about 73% of participants had worked in Vietnam for 3 years or above. Additionally, nearly 56.9% of the MNCs where managers worked had less than 500 employees, and most of these MNCs are located in the north of Vietnam (45.2%).

## Results

In this study, to test the direct and indirect relationships, we employed structural equation modeling (SEM) using the SPSS and Smart PLS 3.0. We adopted Smart PLS structural equation modeling. The application of the SEM in this study has the following two advantages. First, the reason is the structural model is complex and contains a series of dependent relationships (Ringle et al., 2015). Second, the attitude, behavior, and other variables cannot be simply measured with a single item, and certain errors exist. At the same time, the SEM can allow independent and dependent variables to contain measurement errors.

### Common Method Variance

Given our research used self-reported data, we adopted the single-factor approach to test common method variance (Podsakoff et al., 2003). Exploratory factor analysis indicated that the first factor explained only 34.145% of the variance, which was lower than the 50% threshold. Therefore, we argued that no significant common method variance existed as the value was within the acceptable range.

### Reliability and Validity

We used Cronbach’s alpha and composite reliability (CR) to examine the reliability. As illustrated in Table 1, Cronbach’s α values for each dimension ranged from 0.879 to 0.938 (CC, respectively), and CR ranged from 0.904 to 0.946 (CC, respectively). Furthermore, rho_A, a new indicator coefficient introduced in the Smart PLS3.0, was evaluated to rectify the estimate of the measured structure, which ranged from 0.885 to 0.939 (CC, respectively) was well above the recommended threshold of 0.7. These results indicated that the internal consistency of our measurement was at a satisfactory level (Hair et al., 2017).

**TABLE 1:** Confirmatory factor analysis.

Construct	Items	Factor loading	A	rho_A	CR	AVE	VIF
LL	LL1	0.714	0.879	0.885	0.904	0.543	1.446
	LL2	0.667					
	LL3	0.748					
	LL4	0.664					
	LL5	0.677					
	IL6	0.809					
	LL7	0.812					
	LL8	0.785					
CS	CS1	0.713	0.894	0.895	0.913	0.512	DV
	CS2	0.708					
	CS3	0.745					
	CS4	0.719					
	CS5	0.727					
	CS6	0.745					
	CS7	0.718					
	CS8	0.697					
	CS9	0.731					
	CS10	0.651					
CC	CC1	0.700	0.938	0.939	0.946	0.595	1.000
	CC2	0.681					
	CC3	0.743					
	CC4	0.722					
	CC5	0.766					
	CC6	0.715					
	CC7	0.689					
	CC8	0.718					
	CC9	0.690					
	CC10	0.706					
	CC11	0.703					
	CC12	0.721					

*LL, lifelong learning; CC, career competencies; CS, career sustainability.*

Convergent validity can be examined by checking whether the factor loadings of all items were above 0.6 and the average variance extracted (AVE) values were higher than 0.5 (Hair et al., 2017). As shown in Table 1, the factor loadings of all items ranged from 0.651 to 0.812 (CC, respectively), and AVE ranged from 0.512 to 0.595 (CC, respectively). Therefore, our measurement model had an acceptable convergent validity. Moreover, according to Table 1, the variance inflation factor (VIF) values were below 5, indicating no collinearity problems.

In terms of the evaluation of discriminant validity, we referred to the threshold proposed by Fornell and Larcker (1981). The statistics showed that all square roots of the AVE values were larger than the correlation coefficients. The discriminant validity of our measures was confirmed. Results are shown in Table 2.

**TABLE 2:** Discriminant validity analysis (Fornell and Larcker).

Construct	Lifelong learning	Career sustainability	Career competencies
LL	0.737		
CS	0.550	0.716	
CC	0.555	0.702	0.713

*LL, lifelong learning; CC, career competencies; CS, career sustainability.*

### Structural Model and Results Analysis

**FIGURE 2:**
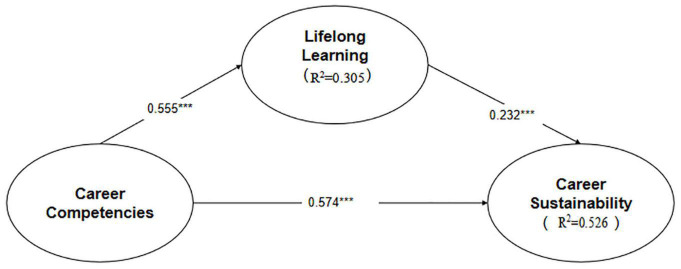
PLS results outcomes. ***t = 1.96.

To test the hypotheses, the bootstrap resampling method in SmartPLS was used to evaluate the PLS results, and the responses were resampled 5,000 times (Hair et al., 2017). Table 3 presents the results. The overall *R*^2^ value was 0.526, indicating that the overall research model accounted for 52.6% of the variance in career sustainability and thus has good explanatory power. The empirical findings supported hypotheses H1, H2, H3, and H4. The results suggested that career competencies are positively related to career sustainability (H1, β = 0.574. *p* < 0.01); career competencies was significantly related to lifelong learning (H2, β = 0.555, *p* < 0.01); lifelong learning was significantly related to career sustainability (H3,β = 0.232, *p* < 0.01). The mediating assumption that lifelong learning mediates the relationship between career competencies and career sustainability was verified (H4, β = 0.129, *p* < 0.001). Figure 2 presents PLS results of the research model.

**TABLE 3:** Serial mediation results.

Hypothesis	Effect	*T*-value	*p*-Value	Result
H1: CC → CS	0.574	9.589	0.001	Significant
H2: CC → LL	0.555	9.484	0.001	Significant
H3: LL → CS	0.232	3.653	0.001	Significant
H4: CC → LL → CS	0.129	3.294	0.001	Significant

*LL, lifelong learning; CC, career competencies; CS, career sustainability.*

## Discussion

Prior studies indicate that career competencies on career sustainability have attracted the attention of many researchers. Previously, such kind of studies were conducted in advanced nations. This is the first study to be conducted in a developing nation like Vietnam. Moreover, it is also the first study to be conducted among the Chinese expatriate managers working at China-invested manufacturing MNCs in Vietnam.

In this article, our results support the linkages among career competencies, lifelong learning, and career sustainability. First, the outcomes of this study indicate that in the direct relationship, career competencies significantly influence career sustainability, which supports H1. A large-scale survey was conducted by Rasool et al. (2019) in the Chinese banking sector, and the findings of their study also support our results. Second, our results also confirmed that career competencies positively and significantly correlate with lifelong learning, which supports H2. Zhou et al. (2021) conducted a study in Chinese small and medium-sized organizations. The outcomes of their study also indicate that career competencies positively impact lifelong learning. Third, the findings of this study confirmed that lifelong learning is significantly and positively associated with career sustainability, which supports our intuition drafted in H3. The results of Samma et al. (2020) also support the outcomes of our study. Fourth, the outcome of this study also testifies the mediating effect of lifelong learning in the relationship between career competencies and career sustainability. Therefore, the results confirmed that lifelong learning has positively mediated career competencies and career sustainability, supporting H4.

### Theoretical Implications

The above-mentioned outcomes have three main useful theoretical implications for researchers and academicians. First, this study enriches the existing literature on sustainable careers by addressing the critical role of lifelong learning through empirical evidence. Previously, most of the research has been conducted from the perspective of “education” instead of “learning.” For instance, some studies defined “lifelong learning” as having the same meaning as “continual education” (Aspin, 2004). Recently, some researchers have focused on the learning perspective, but these studies have been mainly limited to the lifelong learning mindset (Drewery et al., 2020). However, according to the current literature, lifelong learning involves not only a learning mindset but also learning behaviors and capabilities (Hojat et al., 2006). Our research partly fills this gap by positioning lifelong learning as a whole concept, including motivation, ability, and activity where career sustainability is concerned. Second, the current research probes how career competencies enhance career sustainability using lifelong learning as a mediator. This is the first study to be conducted in the Chinese manufacturing industry in Vietnam. Therefore, our findings add value to the cross-cultural working environment. Specifically, to transform the digitalization by the Chinese expatriate managers from a theoretical perspective, previous studies related to this topic were mainly investigated in Western contexts, although recently there has been an increasing volume of research in the Chinese context (Chin et al., 2019a; Ren et al., 2020; Rasool et al., 2021b), there is still a limited range of literature related to developing nations. Therefore, our findings have enriched related research in the cross-cultural setting in developing nations by extending the study scope of career sustainability to the Sino-Vietnamese context.

### Practical Implications

From the perspective of sustainable development of MNCs under the Industry 4.0 revolution, our study suggests some useful implications for the expatriates of the manufacturing industry. Specifically, this study is useful for the mid-level managers who are associated with multinational organizations. Therefore, this study suggests that the mid-level managers who engage in transforming the technologies of multinational organizations need to create a supportive workplace environment. Furthermore, the working environment of expatriates may get more complex due to the cultural differences in the host country. Therefore, this study suggests that the managers need to adopt the local culture of the organizations. From the perspective of international human resource management, our findings suggested that the career competencies of expatriates had a strong impact on their career sustainability in this cross-cultural context. Based on the findings, managers with stronger career competencies, particularly intercultural competencies, should have the prior choice to be expatriated by the MNCs.

### Limitations and Future Research

Our study has given very useful outcomes. Specifically, during COVID-19 it was a big challenge for Chinese expatriates to transform the digitalization in the Vietnam organizations. However, this study had certain limitations. First, the sample size was small. A larger sample size will provide a more diversified sample that ought to be used to test the proposed model in future research for a further extension of the validity of the end results. Second, in this study, we collected data through a self-administrated survey from the Chinese expatriate managers, which may bias the results. Therefore, in the future researchers may conduct research to collect data from direct colleagues, supervisors, or other related stakeholders. Third, in this study, there is no support of any theory. Therefore, future research may also enlarge the present framework by merging resource-based view theory or other performance-based theories. Lastly, most of the lifelong learning measurements were developed in the educational field with students as samples so that the validity of scales applied in our study is limited. Therefore, it is significant to develop valid and reliable lifelong learning measurements under the international workplace context in future discussion.

## Data Availability Statement

The raw data supporting the conclusions of this article will be made available by the authors, without undue reservation.

## Ethics Statement

Ethical review and approval was not required for the study on human participants in accordance with the local legislation and institutional requirements. The participants provided their written informed consent to participate in this study.

## Author Contributions

WZ conceived and designed the research, provided guidance throughout the entire research process, and wrote the main part of the manuscript. FL collected the data and wrote the methods section. TC and C-LL wrote the hypothesis development and methodology sections and offered modification suggestions. Y-NS and FV participated in the online survey and helped analyze the data. All authors listed have made a substantial, direct, and intellectual contribution to the work, and approved it for publication.

## Conflict of Interest

The authors declare that the research was conducted in the absence of any commercial or financial relationships that could be construed as a potential conflict of interest.

## Publisher’s Note

All claims expressed in this article are solely those of the authors and do not necessarily represent those of their affiliated organizations, or those of the publisher, the editors and the reviewers. Any product that may be evaluated in this article, or claim that may be made by its manufacturer, is not guaranteed or endorsed by the publisher.

## Footnotes

1
http://www.vietchina.org/

2
https://www.wjx.cn
